# Food fortification for impact: a data-driven approach

**DOI:** 10.2471/BLT.15.164756

**Published:** 2016-07-05

**Authors:** LM Neufeld, GJ Aaron, GS Garrett, SK Baker, O Dary, M Van Ameringen

**Affiliations:** a Global Alliance for Improved Nutrition, PO Box 55, CH-1211 Geneva, Switzerland.; b Bill & Melinda Gates Foundation, Seattle, United States of America (USA).; c United States Agency for International Development, Washington, USA.

The importance of scientific evidence to guide nutrition policy and programme design is well established.^1^ Nevertheless, there are still critical gaps in the evidence to inform nutrition programme priorities and achievements,^2^ and strong pleas have been made for improving the collection and use of evidence in the nutrition sector.^3^ Nutrition interventions are founded on a strong evidence base from clinical trials, the systematic review of those trials and the translation of this evidence into global guidance.^1^ However, we have far less evidence, particularly for functional outcomes,^4^ on the impact of nutrition programmes that incorporate – but often go beyond – single interventions. We also know less about the pathways by which impacts are achieved in a programmatic context.^5^ Despite the importance of programme coverage for achieving impact, only a few nutrition interventions have sound coverage estimates, including high-dose vitamin A supplementation, household use of iodized salt and, in some countries, community management of acute malnutrition.^2^

In an effort to address these evidence gaps, the Global Nutrition Report of 2014^2^ and the Micronutrient Forum 2014 Global Conference proceedings^6^ specifically highlighted the need to pay more attention to programme coverage as the main approach to assessing the availability, access and utilization of nutrition programmes. Moreover, the World Health Organization, as part of the Comprehensive Implementation Plan on Maternal, Infant and Young Child Nutrition,^7^ has called for renewed and coordinated efforts to better track programme coverage and to build systems for data-driven decision-making. Fortification of staple foods is usually mandated and regulated by governments and, when this functions well, is enforced at the point of production or import. But when foods are then sold on the market, it is difficult for health information or surveillance systems to track their use. Fortification of staple foods by the addition of vitamins and/or minerals to food at processing is widely recognized to be a cost–effective public health intervention that can reach large segments of the population.^8^ For example, goitre and other severe iodine deficiency disorders have been virtually eliminated in most countries around the world due to iodization of salt,^9^ and the contribution of wheat flour fortification with folic acid to reductions in the incidence of neural tube defects^10^ in several countries is well documented. At the same time there is still some controversy around the safety of long-term high intakes of some nutrients, most notably folic acid,^11^ thus again highlighting the need to measure dietary sources of nutrients and to track additional intakes due to the consumption of fortified foods.

While in most countries governments regulate food fortification – i.e. what can be fortified, with which nutrients and at what levels – monitoring of compliance and enforcement of regulations needs to be strengthened considerably in many countries.^12^ Further compounding the issue are the lack of data on dietary intakes in most countries and how dietary patterns vary by population sub-groups – e.g. by socioeconomic status, geographical region or ethnicity. Such information is important to identify the dietary nutrient gaps that need to be addressed, as well as which nutrition strategies may be viable from a programmatic perspective. Data about intakes are required to set appropriate levels of fortificants, as well as to assess the extent to which those in need are actually consuming the foods, with what frequency and quantity.^8^ Some efforts have been made to fill these gaps, via analysis of household income and expenditure data at the national level,^13^ but the lack of detail about the types of food consumed and individuals’ consumption levels may limit the utility of these data to fully inform food fortification programmes.^14^ The Fortification Rapid Assessment Tool (FRAT)^15^ and more recently the Fortification Assessment Coverage Tool (FACT)^16^ were designed to provide more comprehensive information on consumption of fortified foods and foods that could potentially be fortified. Indicators of the use of fortified foods, and even food sample collection (e.g. salt samples) to verify compliance, have been incorporated into national surveillance systems in a few countries – e.g. Nicaragua^17^ – and modules from FRAT or FACT could potentially be adapted and included as well.

The big question remains: how to garner the resources and political commitment needed for the generation and use of evidence for programme decision-making in nutrition broadly and food fortification specifically? This question was high on the agenda at the first Global Summit on Food Fortification in Arusha, United Republic of Tanzania from 9 to 11 September 2015. Government delegations from 29 countries from Africa, Asia and Central and South America, and experts and representatives from donors, United Nations agencies, nongovernmental organizations working in food fortification and the private sector, met to discuss and debate the state of food fortification in their countries and globally. The Summit declaration^18^ highlighted the need for increased investment and use of evidence to inform fortification programmes as part of national nutrition strategies. We hope that one of the lasting outcomes of this meeting will be greater commitment from countries and donors for evidence-driven decision-making on food fortification, as part of micronutrient deficiency control strategies – where it is needed and where it can have impact.

## References

[1] Neufeld LM, Jalal CS, Peña-Rosas JP, Tovey D, Lutter CK, Stoltzfus RJ, et al. The WHO evidence-informed guideline development process and implications for vitamin and mineral research priorities: symposium rationale and summary. Adv Nutr. 2013 9 1;4(5):557–9. 10.3945/an.113.004291 24038256 PMC3771148

[2] Global nutrition report 2014: actions and accountability to accelerate the world’s progress on nutrition. Washington: International Food Policy Research Institute; 2014. Available from: http://cdm15738.contentdm.oclc.org/utils/getfile/collection/p15738coll2/id/128484/filename/128695.pdf [cited 2015 Aug 5]. 10.3945/an.115.008599 PMC4424785 25979494

[3] A miracle cure for global malnutrition? The data diet. Twenty-fourth annual Martin J Forman memorial lecture [Internet]. Washington: International Food Policy Research Institute; 2014. Available from: http://www.ifpri.org/event/24th-annual-martin-j-forman-memorial-lecture [cited 2016 Jun 15].

[4] Bhutta ZA, Das JK, Rizvi A, Gaffey MF, Walker N, Horton S, et al.; Lancet Nutrition Interventions Review Group; Maternal and Child Nutrition Study Group. Evidence-based interventions for improvement of maternal and child nutrition: what can be done and at what cost? Lancet. 2013 8 3;382(9890):452–77. 10.1016/S0140-6736(13)60996-4 23746776

[5] Habicht JP, Pelto GH. From biological to program efficacy: promoting dialogue among the research, policy, and program communities. Adv Nutr. 2014 Jan 1;5(1):27–34. 10.3945/an.113.004689 24425719 PMC3884096

[6] Bridging discovery and delivery. Micronutrient Forum Global Conference. Addis Ababa, Ethiopia, 2–6 June 2014 [Internet]. Ottawa: Micronutrient Forum; 2014. Available from: http://micronutrientforum.org/content/user_files/2016/01/Micronutrient-Forum-Global-Conference-Ethiopia-2014-Proceedings.pdf [cited 2016 Jun 15].

[7] Comprehensive implementation plan on maternal, infant and young child nutrition. Geneva: World Health Organization; 2014. Available from: http://apps.who.int/iris/bitstream/10665/113048/1/WHO_NMH_NHD_14.1_eng.pdf [cited 2015 Aug 5]. 10.3945/an.114.007781 PMC4288273 25593153

[8] Guidelines on food fortification with micronutrients. Geneva: World Health Organization; 2006. Available from: http://www.who.int/nutrition/publications/guide_food_fortification_micronutrients.pdf [cited 2015 Aug 5].

[9] Zimmermann MB. Iodine deficiency and excess in children: worldwide status in 2013. Endocr Pract. 2013 Sep-Oct;19(5):839–46. 10.4158/EP13180.RA 23757630

[10] Castillo-Lancellotti C, Tur JA, Uauy R. Impact of folic acid fortification of flour on neural tube defects: a systematic review. Public Health Nutr. 2013 5;16(5):901–11. 10.1017/S1368980012003576 22850218 PMC10271422

[11] Choi JH, Yates Z, Veysey M, Heo YR, Lucock M. Contemporary issues surrounding folic acid fortification initiatives. Prev Nutr Food Sci. 2014 12;19(4):247–60. 10.3746/pnf.2014.19.4.247 25580388 PMC4287316

[12] Luthringer CL, Rowe LA, Vossenaar M, Garrett GS. Regulatory monitoring of fortified foods: identifying barriers and good practices. Glob Health Sci Pract. 2015 9 02;3(3):446–61. 10.9745/GHSP-D-15-00171 26374804 PMC4570017

[13] Fiedler JL, Martin-Prével Y, Moursi M. Relative costs of 24-hour recall and household consumption and expenditures surveys for nutrition analysis. Food Nutr Bull. 2013 9;34(3):318–30. 10.1177/156482651303400304 24167912

[14] Engle-Stone R, Brown KH. Comparison of a household consumption and expenditures survey with nationally representative food frequency questionnaire and 24-hour dietary recall data for assessing consumption of fortifiable foods by women and young children in Cameroon. Food Nutr Bull. 2015 6;36(2):211–30. 10.1177/0379572115587272 26121703

[15] Fortification rapid assessment tool and guidelines [Internet]. Cincinnati: HealthBridge; 2003. Available from: http://www.micronutrient.org/nutritiontoolkit/ModuleFolders/3.Indicators%5CDietary%5CTools%5CFortification_Rapid_Assessment_Tool_and_Guidelines.pdf [cited 2016 Jun 24].

[16] Aaron G, Siling K, Norris A, Guevarra E, Myatt M. Assessing coverage of targeted and large-scale fortification programs: development of a fortification assessment coverage tool (FACT) [conference abstract] Eur J Nutr Food Saf. 2015;5(5):932–3. 10.9734/EJNFS/2015/21171

[17] Sistema integrado de vigilancia de intervenciones nutricionales (SIVIN) [Internet]. Guatemala: Instituto de Nutrición de Centroamérica y Panamá; 2004. Available from: http://www.incap.org.gt/sisvan/index.php/es/areas-tematicas/metodologias-de-apoyo/sistema-integrado-de-vigilancia-de-intervenciones-nutricionales-sivin [cited 2015 Aug 5]. Spanish.

[18] The Arusha statement on food fortification [Internet]. Geneva: Global Alliance for Improved Nutrition; 2015. Available from: http://www.gainhealth.org/wp-content/uploads/2015/05/Arusha-Statement.pdf [cited 2016 Jun 19].

